# Discovery of New Eunicellins from an Indonesian Octocoral *Cladiella* sp.

**DOI:** 10.3390/md9060934

**Published:** 2011-05-26

**Authors:** Yung-Husan Chen, Chia-Ying Tai, Yin-Di Su, Yu-Chia Chang, Mei-Chin Lu, Ching-Feng Weng, Jui-Hsin Su, Tsong-Long Hwang, Yang-Chang Wu, Ping-Jyun Sung

**Affiliations:** 1 National Museum of Marine Biology and Aquarium, Pingtung 944, Taiwan; E-Mails: tony_chen72001@yahoo.com.tw (Y.-H.C.); j19851214@hotmail.com (C.-Y.T.); gobetter04@yahoo.com.tw (Y.-D.S.); jay0404@gmail.com (Y.-C.C.); jinx6609@nmmba.gov.tw (M.-C.L.); x2219@nmmba.gov.tw (J.-H.S.); 2 Graduate Institute of Marine Biotechnology, National Dong Hwa University, Pingtung 944, Taiwan; 3 Department of Marine Biotechnology and Resources, National Sun Yat-sen University, Kaohsiung 804, Taiwan; 4 Doctoral Degree Program in Marine Biotechnology, National Sun Yat-sen University and Academia Sinica, Kaohsiung 804, Taiwan; 5 Department of Life Science and Institute of Biotechnology, National Dong Hwa University, Hualien 974, Taiwan; E-Mail: cfweng@mail.ndhu.edu.tw (C.-F.W.); 6 Division of Marine Biotechnology, Asia-Pacific Ocean Research Center, National Sun Yat-sen University, Kaohsiung 804, Taiwan; 7 Graduate Institute of Natural Products, Chang Gung University, Taoyuan 333, Taiwan; E-Mail: htl@mail.cgu.edu.tw (T.-L.H.); 8 Graduate Institute of Integrated Medicine, College of Chinese Medicine, China Medical University, Taichung 404, Taiwan; 9 Natural Medicinal Products Research Center, China Medical University Hospital, Taichung 404, Taiwan

**Keywords:** cladieunicellin, eunicellin, octocoral, *Cladiella*, superoxide anion, elastase

## Abstract

Two new 11-hydroxyeunicellin diterpenoids, cladieunicellin F (**1**) and (–)-solenopodin C (**2**), were isolated from an Indonesian octocoral *Cladiella* sp. The structures of eunicellins **1** and **2** were established by spectroscopic methods, and eunicellin **2** was found to be an enantiomer of the known eunicellin solenopodin C (**3**). Eunicellin **2** displayed inhibitory effects on the generation of superoxide anion and the release of elastase by human neutrophils. The previously reported structures of two eunicellin-based compounds, cladielloides A and B, are corrected in this study.

## 1. Introduction

**Figure 1:**
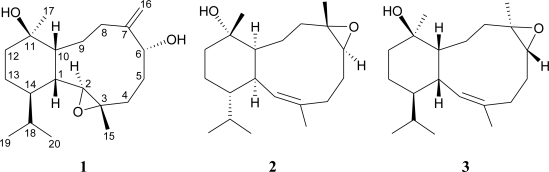
The structures of cladieunicellin F (**1**), (−)-solenopodin C (**2**), and solenopodin C (**3**).

As part of our ongoing investigation into the isolation of new natural substances from octocorals collected in the tropical West Pacific Ocean, a series of interesting diterpenoids, including cembrane-type [1], eunicellin-type (2,11-cyclized cembranoid) [2–4], and briarane-type (3,8-cyclized cembranoid) [5–7] diterpenoids, were isolated from various octocorals belonging to the genera *Briareum*, *Cladiella*, *Ellisella*, and *Junceella*. The chemical constituents of an Indonesian octocoral identified as *Cladiella* sp. were examined, and nine new eunicellin-based diterpenoids, including cladieunicellins A–E [2] and cladielloides A–D [3,4], were isolated. Further study of this coral yielded two new eunicellins, cladieunicellin F (**1**) and (–)-solenopodin C (**2**). In this paper, we report the isolation, structure determination, and bioactivity of new eunicellins **1** and **2** (Figure 1).

## 2. Results and Discussion

Cladieunicellin F (**1**) was isolated as a colorless oil and the molecular formula for this compound was determined using HRESIMS to be C_20_H_34_O_3_ (four degrees of unsaturation) (*m/z* 345.2404 [M + Na]^+^, calculated for 345.2406). Comparison of the ^13^C NMR and DEPT data with the molecular formula indicated that there must be two exchangeable protons, which required the presence of two hydroxyl groups. This deduction was supported by a broad absorption in the IR spectrum at 3414 cm^−1^. The ^13^C NMR data for **1** confirmed the presence of twenty carbon signals (Table 1), which were characterized by DEPT as four methyls, an sp^2^ methylene, six sp^3^ methylenes, six sp^3^ methines (including two oxymethines), two sp^3^ oxygenated quaternary carbons, and an sp^2^ quaternary carbon. Based on the ^1^H and ^13^C NMR spectra (Table 1), **1** was determined to possess an exocyclic carbon-carbon double bond (*δ*_H_ 5.06, 2H, br s, H_2_-16; *δ*_C_ 150.6, s, C-7; 111.8, t, C-16). The presence of a trisubstituted epoxide containing a methyl substituent was established from the signals of an oxygenated quaternary carbon (*δ*_C_ 62.9, s, C-3) and an oxymethine (*δ*_H_ 2.81, 1H, d, *J* = 9.6 Hz; *δ*_C_ 64.8, d, CH-2) and confirmed by the proton signal of a methyl singlet at *δ*_H_ 1.36 (3H, s, H_3_-15). In the ^1^H NMR spectrum of **1**, two doublets at *δ*_H_ 0.88 and 0.81 (each 3H, d, *J* = 6.4 Hz, H_3_-19 and H_3_-20) were indicative of the two methyls of an isopropyl group. A tertiary methyl group bonded to an oxygenated carbon was evident from the singlet signal at *δ*_H_ 1.24 (3H, s, H_3_-17). Thus, from the reported data, the proposed skeleton of **1** was suggested to be an eunicellin-based diterpenoid with three rings.

From the ^1^H–^1^H COSY spectrum of **1** (Table 1), it was possible to differentiate among the separate spin systems of H-1/H-2, H_2_-4/H_2_-5/H-6, H_2_-8/H_2_-9/H-10/H-1, H_2_-12/H_2_-13/H-14/H-1, and H-14/H-18/H_3_-19 (H_3_-20), which was accomplished with the assistance of an HMBC experiment (Table 1). The key HMBC correlations between the protons and quaternary carbons of **1**, including H-1, H-2, H_2_-4, H_2_-5, H_3_-15/C-3; H_2_-5, H-8b, H-9b, H_2_-16/C-7; and H-1, H-10, H_2_-12, H_2_-13, H_3_-17/C-11, permitted the elucidation of the carbon skeleton. An exocyclic carbon-carbon double bond at C-7 was confirmed by the HMBC correlations between H_2_-16/C-6, -7, -8; H-6/C-16; and H-8b/C-16 and further supported by the allylic coupling between H-8b/H_2_-16. The presence of the C-2/3 epoxide group was confirmed by the HMBC correlations between H-1/C-2, -3; H-2/C-1, -3, -10, -15; H_2_-4/C-2, -3; and H_3_-15/C-2, -3, -4. Thus, the remaining hydroxyl groups should be positioned at C-6 and C-11, as indicated by the key ^1^H–^1^H COSY correlations and characteristic NMR signals, although the hydroxyl protons for OH-6 and OH-11 were not observed in the ^1^H NMR spectrum of **1**.

**Figure 2:**
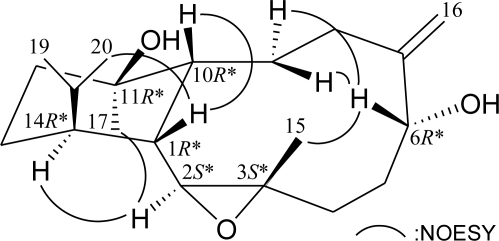
Selective NOESY correlations for **1**.

The relative configuration of **1** was elucidated from the interactions observed in a NOESY experiment (Figure 2): H-1 correlated with H-10 and H_3_-20, indicating that H-1, H-10, and the isopropyl group are situated on the same face and assigned as β protons. H-2 exhibited interactions with H-14 and Me-17, and no correlation was found between H-1/H-2, H-10/Me-17, and H-2/Me-15, indicating that H-2, H-14, and Me-17 should be α-oriented and Me-15 should be β-oriented. Furthermore, H-6 correlated with two protons of the C-9 methylene and Me-15. Consideration of molecular models found that H-6 was reasonably close to H_2_-9 and Me-15 when H-6 was β-oriented in **1**. Based on the above findings, the structure of **1**, including its relative configuration, was established, and the chiral centers for **1** were assigned as 1R*, 2S*, 3S*, 6R*, 10R*, 11R*, and 14R*. To the best of our knowledge, cladieunicellin F (**1**) is the first eunicellin derivative possessing a C-2/3 epoxy group.

Eunicellin **2** was isolated as a colorless oil, and the molecular formula of this compound was determined using HRESIMS to be C_20_H_34_O_2_ (*m/z* 329.2455 [M + Na]^+^, calculated for 329.2456). Thus, four degrees of unsaturation were determined for **2**. Detailed analysis of the NMR data showed that the data for **2** were similar to those of a known eunicellin analogue, solenopodin C (**3**) (Figure 1), which was isolated from the gorgonian *Solenopodium stechei* [8]. However, the optical rotation value of **2** ( ${\left[\alpha \right]}_{\text{D}}^{22}$ −51 (*c* 0.17, CHCl_3_)) was substantially different from that of **3** ( ${\left[\alpha \right]}_{\text{D}}^{22}$ +105.6 (*c* 0.36)), indicating that eunicellin **2** is an enantiomer of **3** and should be designated (–)-solenopodin C. The ^1^H and ^13^C NMR data for **2** (Table 2) were assigned using 2D NMR data analysis and comparison to the NMR data of **3**. The proton chemical shifts for C-8, C-9, C-12, and C-13 methylene protons and the carbon chemical shifts for C-1, C-4, C-12, and C-14 of compound **3** should be revised (Table 2).

**Figure 3:**
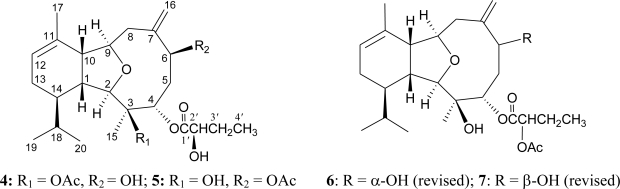
The previously reported structures of cladielloides A (**4**) and B (**5**) and their revised structures **6** and **7**, respectively.

In a previous study, we reported the isolation and structure determination of two eunicellins, cladielloides A (**4**) and B (**5**) (Figure 3) [3]. However, based on detailed spectral data analysis, we found that the structures for these two compounds should be revised. 1D and 2D NMR spectral data analysis, particularly ^1^H–^1^H COSY and HMBC experiments, of cladielloide A (Table 3), showed that the main carbon skeleton of cladielloide A was established correctly. However, in the HMBC experiment for cladielloide A, key correlations between H-4 (*δ*_H_ 5.14) and an ester carbonyl at *δ*_C_ 171.4 (s, C-1′) and between H-2′ (*δ*_H_ 4.86) and two ester carbonyls at *δ*_C_ 171.4 (s, C-1′) and 171.1 (s, acetate carbonyl) were detected, and these findings indicated that the 2′-acetoxybutyrate group should be positioned at C-4. Thus, the remaining hydroxyl groups are attached at C-3 and C-6 in cladielloide A, respectively. Furthermore, in the NOESY spectrum of cladielloide A, H-6 (*δ*_H_ 4.21) correlated with H_2_-5 (*δ*_H_ 2.97 and 1.75) and H_2_-8 (*δ*_H_ 2.35), but no correlation was found between H-6 and H_3_-15. Consideration of molecular models found that H-6 was reasonably close to H_2_-5 and H_2_-8 when it was β-oriented. Based on the above findings, the structure, including the relative configuration, of cladielloide A should be revised as presented in eunicellin **6**. Cladielloide B was found by HRESIMS to be an isomer of cladielloide A [3]. These two compounds were found to possess the same planar structure by NMR data analysis (Tables 3 and 4). In the NOESY experiment of cladielloide B, H-6 (*δ*_H_ 4.66) exhibited correlations with H_3_-15 (*δ*_H_ 1.33); a proton of C-5 methylene (*δ*_H_ 2.48); and a proton of C-8 methylene (*δ*_H_ 2.65), indicating that the 6-hydroxyl group in cladielloide B should be β-oriented as presented as eunicellin **7**. Based on the above findings, the structures of cladielloides A and B should be revised as structures **6** and **7**, respectively. The authors apologize for any inconvenience caused by these errors.

The *in vitro* anti-inflammatory effects of eunicellins **1** and **2** were tested. Eunicellin **2** displayed significant inhibitory effects on the generation of superoxide anion and the release of elastase by human neutrophils at a concentration of 10 μg/mL (Table 5).

## 3. Experimental Section

### 3.1. General Experimental Procedures

Optical rotation values were measured with a JASCO P-1010 digital polarimeter. Infrared spectra were obtained on a VARIAN DIGLAB FTS 1000 FT-IR spectrophotometer. The NMR spectra were recorded on a VARIAN MERCURY PLUS 400 FT-NMR at 400 MHz and 100 MHz for ^1^H and ^13^C spectra, respectively, in CDCl_3_ at 25 °C. Proton chemical shifts were referenced to the residual CHCl_3_ signal (*δ*_H_ 7.26 ppm). ^13^C NMR spectra were referenced to the center peak of CDCl_3_ at *δ*_C_ 77.1 ppm. ESIMS and HRESIMS data were recorded on a BRUKER APEX II mass spectrometer. Column chromatography was performed on silica gel (230–400 mesh, Merck, Darmstadt, Germany). TLC was carried out on precoated Kieselgel 60 F_254_ (0.25 mm, Merck), and spots were visualized by spraying with 10% H_2_SO_4_ solution followed by heating. HPLC was performed using a system comprised of a HITACHI L-2130 pump, a HITACHI photodiode array detector L-2455, and a RHEODYNE 7725 injection port. A reverse phase column (Polaris 5 C18-A 250 × 10.0 mm, Varian, silica gel 60, 5 μm) was used for HPLC.

### 3.2. Animal Material

The octocoral *Cladiella* sp. was collected and imported legitimately by the National Museum of Marine Biology and Aquarium (NMMBA), Taiwan from Indonesia in 2004. The material was stored in a freezer until extraction procedures were applied. A voucher specimen (NMMBA-IND-SC-001) was deposited in the NMMBA, Taiwan. This organism was identified by comparison with previous descriptions [10,11].

### 3.3. Extraction and Isolation

Sliced bodies of *Cladiella* sp. (wet weight, 924 g) were extracted with a mixture of MeOH and CH_2_Cl_2_ (1:1), and the residue collected after solvent evaporation was partitioned between EtOAc and H_2_O. The EtOAc layer was subjected to silica gel column chromatography and eluted using a mixture of *n*-hexane and EtOAc (stepwise from 100:1 to 0:100 *n*-hexane:EtOAc) to obtain 19 fractions, labeled A–S. Fractions F and I were separated by reverse phase HPLC using a mixture of MeOH and water to afford eunicellins **2** (3.3 mg, 1/1) and **1** (1.5 mg, 1/1), respectively.

Cladieunicellin F (**1**): colorless oil; ${\left[\alpha \right]}_{\text{D}}^{23}$ −194 (*c* 0.07, CHCl_3_); IR (neat) ν_max_ 3414 cm^−1; 1^H NMR (CDCl_3_, 400 MHz) and ^13^C NMR (CDCl_3_, 100 MHz) data, see Table 1; ESIMS *m/z* 345 [M + Na]^+^; HRESIMS *m/z* 345.2404 (Calcd for 345.2406).

(–)-Solenopodin C (**2**): colorless oil; ${\left[\alpha \right]}_{\text{D}}^{22}$ −51 (*c* 0.17, CHCl_3_); IR (neat) ν_max_ 3427 cm^−1; 1^H NMR (CDCl_3_, 400 MHz) and ^13^C NMR (CDCl_3_, 100 MHz) data, see Table 2; ESIMS *m/z* 329 [M + Na]^+^; HRESIMS *m/z* 329.2455 (Calcd for 329.2456).

### 3.4. Superoxide Anion Generation and Elastase Release by Human Neutrophils

Human neutrophils were obtained using dextran sedimentation and Ficoll centrifugation. Measurements of superoxide anion generation and elastase release were carried out according to previously described procedures [12,13]. Briefly, superoxide anion production was assayed by monitoring the superoxide dismutase-inhibitable reduction of ferricytochrome *c*. Elastase release experiments were performed using MeO-Suc-Ala-Ala-Pro-Valp-nitroanilide as the elastase substrate.

## Figures and Tables

**Table 1:** ^1^H and ^13^C NMR data, ^1^H–^1^H COSY, and HMBC correlations for diterpenoid **1**.

**C/H**	**^1^H^a^**	**^13^C^b^**	**^1^H–^1^H COSY**	**HMBC (H→C)**
1	2.12 m	36.0 (d)^d^	H-2, H-10, H-14	C-2, C-3, C-9, C-10, C-11, C-14, C-18
2	2.81 d (9.6)^c^	64.8 (d)	H-1	C-1, C-3, C-10, C-15
3		62.9 (s)		
4a	1.27 m	27.3 (t)	H-4b, H_2_-5	C-3, C-5, C-15
b	1.81 m		H-4a, H_2_-5	C-2, C-3, C-5, C-6, C-15
5	2.01 m	30.3 (t)	H_2_-4, H-6	C-3, C-4, C-6, C-7
6	4.20 dd (10.4, 4.0)	68.4 (d)	H_2_-5	C-16
7		150.6 (s)		
8a	2.16 m	37.5 (t)	H-8b, H_2_-9	n.o.^e^
b	2.61 m		H-8a, H_2_-9, H_2_-16	C-7, C-9, C-10, C-16
9a	1.44 m	22.9 (t)	H_2_-8, H-9b, H-10	n.o.
b	1.96 m		H_2_-8, H-9a, H-10	C-1, C-7, C-8
10	1.68 dd (10.8, 6.0)	46.1 (d)	H-1, H_2_-9	C-1, C-2, C-8, C-9, C-11, C-12, C-17
11		72.9 (s)		
12	1.59 m	37.2 (t)	H_2_-13	C-10, C-11, C-13, C-14, C-17
13a	1.55 m	21.8 (t)	H_2_-12, H-13b, H-14	C-11, C-12, C-14
b	1.71 m		H_2_-12, H-13a, H-14	C-11, C-12, C-14
14	1.32 m	41.5 (d)	H-1, H_2_-13, H-18	C-13
15	1.36 s	21.8 (q)		C-2, C-3, C-4
16	5.06 br s	111.8 (t)	H-8b	C-6, C-7, C-8
17	1.24 s	24.4 (q)		C-10, C-11, C-12
18	1.81 m	26.5 (d)	H-14, H_3_-19, H_3_-20	C-14, C-19, C-20
19	0.88 d (6.4)	20.6 (q)	H-18	C-14, C-18, C-20
20	0.81 d (6.4)	21.7 (q)	H-18	C-14, C-18, C-19

a Spectra were measured at 400 MHz in CDCl_3_.

b Spectra were measured at 100 MHz in CDCl_3_.

c *J* values (in hertz) are in parentheses.

d Attached protons were deduced by DEPT and HMQC experiments.

e n.o. = not observed.

**Table 2:** ^1^H and ^13^C NMR data for diterpenoids **2** and **3**.

**C/H**	**2**		**3**	
	**^1^H^a^**	**^13^C^b^**	**^1^H^e^**	**^13^C^e^**
1	2.73 ddd (7.6, 6.0, 6.0)^c^	36.6 (d)^d^	2.73 ddd (7.5, 6, 6)	44.8 (d)
2	5.35 d (7.6)	130.4 (d)	5.34 br d (7.5)	130.4 (d)
3		134.3 (s)		134.3 (s)
4a	2.47 ddd (13.2, 13.2, 2.8)	28.0 (t)	2.47 ddd (13.4, 13, 2.2)	35.2 (t)
b	1.95 m		1.9 m	
5a	2.13 m	25.3 (t)	2.1 m	25.3 (t)
b	1.37 m		1.3 m	
6	3.10 dd (10.8, 3.6)	65.9 (d)	3.10 dd (11.2, 3.5)	65.9 (d)
7		61.2 (s)		61.1 (s)
8a	2.14 m	38.6 (t)	2.3 m	38.6 (t)
b	0.93 m			
9a	1.58 m	22.5 (t)	1.2–1.7 m	22.2 (t)
b	0.90 m			
10	1.95 m	47.3 (d)	1.95 ddd (6, 6, 6)	47.3 (d)
11		73.2 (s)		73.2 (s)
12a	1.52 m	35.2 (t)		28.1 (t)
b	1.49 m			
13a	1.56 m	20.0 (t)		20.0 (t)
b	1.45 m			
14	1.02 m	44.7 (d)	1.0 m	36.6 (d)
15	1.71 s	24.9 (q)	1.71 s	24.9 (q)
16	1.18 s	18.2 (q)	1.22 s	18.2 (q)
17	1.22 s	26.7 (q)	1.18 s	26.8 (q)
18	1.95 m	26.6 (d)	1.90 m	26.6 (d)
19	0.97 d (6.8)	22.0 (q)	0.78 d (7)	22.0 (q)
20	0.78 d (6.8)	17.6 (q)	0.98 d (7)	17.7 (q)

a Spectra were measured at 400 MHz in CDCl_3_.

b Spectra were measured at 100 MHz in CDCl_3_.

c *J* values (in hertz) are in parentheses.

d Attached protons were deduced by DEPT and HMQC experiments.

e Data were reported by Bloor et al. [8]. These data were measured at 300 MHz for ^1^H and 75 MHz for ^13^C in CDCl_3_.

**Table 3:** ^1^H and ^13^C NMR data, ^1^H–^1^H COSY, and HMBC correlations for cladielloide A (**6**).

**C/H**	**^1^H^a^**	**^13^C^b^**	**^1^H–^1^H COSY**	**HMBC (H→C)**
1	2.74 ddd (8.0, 8.0, 4.0)^c^	39.7 (d)^d^	H-2, H-10, H-14	C-2, C-3, C-10, C-11, C-14
2	3.86 d (8.0)	87.1 (d)	H-1	C-3, C-4, C-14, C-15
3		74.1 (s)		
4	5.14 dd (4.4, 4.4)	74.6 (d)	H_2_-5	C-3, C-6, C-15, C-1′
5α	2.97 ddd (16.0, 4.4, 2.8)	37.2 (t)	H-4, H-5β, H-6	C-3
β	1.75 ddd (16.0, 5.6, 3.6)		H-4, H-5α, H-6	C-3, C-4, C-7
6	4.21 br s	72.6 (d)	H_2_-5, OH-6	n.o.^e^
7		147.6 (s)		
8	2.35 br d (2.4)	40.0 (t)	H-9	C-6, C-7, C-9, C-10, C-16
9	4.16 ddd (3.6, 3.2, 3.2)	81.3 (d)	H_2_-8, H-10	n.o.
10	2.63 br s	44.6 (d)	H-1, H-9	C-11
11		132.1 (s)		
12	5.43 m	122.2 (d)	H_2_-13, H_3_-17	n.o.
13α	2.10 m	22.8 (t)	H-12, H-13β, H-14	n.o.
β	1.98 m		H-12, H-13α, H-14	n.o.
14	1.58 m	39.0 (d)	H-1, H_2_-13, H-18	n.o.
15	1.37 s	22.4 (q)		C-2, C-3, C-4
16a	5.21 s	115.2 (t)	H-16b	C-6, C-8
b	5.58 s		H-16a	C-6, C-7, C-8
17	1.68 d (0.8)	22.0 (q)	H-12	
18	1.15 m	28.8 (d)	H-14, H_3_-19, H_3_-20	
19	0.92 d (6.4)	21.3 (q)	H-18	C-14, C-18, C-20
20	0.83 d (6.4)	20.5 (q)	H-18	C-14, C-18, C-19
OH-6	2.84 d (7.2)		H-6	n.o.
1′		171.4 (s)		
2′	4.86 dd (6.8, 6.0)	74.4 (d)	H_2_-3′	C-1′, C-3′, C-4′, acetate carbonyl
3′	1.91 m	24.3 (t)	H-2′, H_3_-4′	C-1′, C-2′, C-4′
4′	1.03 t (7.2)	9.3 (q)	H_2_-3′	C-2′, C-3′
2′-OAc		171.1 (s)		
	2.14 s	20.6 (q)		Acetate carbonyl

a Spectra were measured at 400 MHz in CDCl_3_.

b Spectra were measured at 100 MHz in CDCl_3_.

c *J* values (in hertz) are in parentheses.

d Attached protons were deduced by DEPT and HMQC experiments.

e n.o. = not observed.

**Table 4:** ^1^H and ^13^C NMR data, ^1^H–^1^H COSY, and HMBC correlations for cladielloide B (**7**).

**C/H**	**^1^H^a^**	**^13^C^b^**	**^1^H–^1^H COSY**	**HMBC (H**→**C)**
1	2.51 m	40.6 (d)^d^	H-2, H-10, H-14	C-10
2	3.90 d (3.6)^c^	88.1 (d)	H-1	C-1, C-3, C-4, C-10
3		74.8 (s)		
4	5.21 dd (8.0, 4.0)	73.8 (d)	H_2_-5	C-5, C-6, C-1′
5α	2.48 m	34.2 (t)	H-4, H-5β, H-6	C-6, C-7
β	1.97 m		H-4, H-5α, H-6	n.o.^e^
6	4.66 dd (8.8, 3.2)	83.8 (d)	H_2_-5	C-4, C-7, C-16
7		144.2 (s)		
8α	2.65 dd (14.0, 4.8)	41.4 (t)	H-8β, H-9, H-16a	C-7, C-9, C-10, C-16
β	2.46 dd (14.0, 2.0)		H-8α, H-9	C-6, C-7, C-16
9	4.06 br s	82.4 (d)	H_2_-8, H-10	n.o.
10	2.58 br s	44.7 (d)	H-1, H-9	C-8, C-9, C-11
11		131.1 (s)		
12	5.49 m	123.1 (d)	H_2_-13, H_3_-17	n.o.
13α	2.01 m	22.9 (t)	H-12, H-13β, H-14	n.o.
β	1.80 m		H-12, H-13α, H-14	n.o.
14	1.39 m	39.8 (d)	H-1, H_2_-13, H-18	C-1, C-2
15	1.33 s	22.8 (q)		C-2, C-3, C-4
16a	5.26 s	117.7 (t)	H-8α, H-16b	C-6, C-8
b	5.47 s		H-16a	C-6, C-7, C-8
17	1.69 d (1.2)	22.8 (q)	H-12	C-10, C-11, C-12
18	1.80 m	27.8 (d)	H-14, H_3_-19, H_3_-20	C-14, C-19, C-20
19	0.94 d (6.8)	21.7 (q)	H-18	C-14, C-18, C-20
20	0.77 d (6.8)	17.5 (q)	H-18	C-14, C-18, C-19
1′		170.2 (s)		
2′	4.87 dd (6.8, 6.0)	74.3 (d)	H_2_-3′	C-1′, C-3′, C-4′, acetate carbonyl
3′	1.91 m	24.5 (t)	H-2′, H_3_-4′	C-1′, C-2′, C-4′
4′	1.02 t (7.2)	9.3 (q)	H_2_-3′	C-2′, C-3′
2′-OAc		171.6 (s)		
	2.14 s	20.6 (q)		Acetate carbonyl

a Spectra were measured at 400 MHz in CDCl_3_.

b Spectra were measured at 100 MHz in CDCl_3_.

c *J* values (in hertz) are in parentheses.

d Attached protons were deduced by DEPT and HMQC experiments.

e n.o. = not observed.

**Table 5:** Inhibitory effects of eunicellins **1** and **2** on the generation of superoxide anion and the release of elastase by human neutrophils in response to FMLP/CB.

	**Superoxide anion**	**Elastase release**
**Compounds**	**Inh%**	**Inh%**
**1**	6.46 ± 1.28 **	12.91 ± 3.56 *
**2**	45.82 ± 2.49 ***	40.45 ± 5.80 **

Percentage of inhibition (Inh%) at 10 μg/mL concentration of **1** and **2**. Results are presented as the mean + S.E.M. (*n* = 3).

* *P* < 0.05,

** *P* < 0.01,

*** *P* < 0.001, as compared with the control value [9].
